# Thermo-tunable hybrid photonic crystal fiber based on solution-processed chalcogenide glass nanolayers

**DOI:** 10.1038/srep31711

**Published:** 2016-08-19

**Authors:** Christos Markos

**Affiliations:** 1 DTU Fotonik, Department of Photonics Engineering, Technical University of Denmark, DK-2800 Kgs, Lyngby, Denmark

## Abstract

The possibility to combine silica photonic crystal fiber (PCF) as low-loss platform with advanced functional materials, offers an enormous range of choices for the development of fiber-based tunable devices. Here, we report a tunable hybrid silica PCF with integrated As_2_S_3_ glass nanolayers inside the air-capillaries of the fiber based on a solution-processed glass approach. The deposited high-index layers revealed antiresonant transmission windows from ~500 nm up to ~1300 nm. We experimentally demonstrate for the first time the possibility to thermally-tune the revealed antiresonances by taking advantage the high thermo-optic coefficient of the solution-processed nanolayers. Two different hybrid fiber structures, with core diameter 10 and 5 μm, were developed and characterized using a supercontinuum source. The maximum sensitivity was measured to be as high as 3.6 nm/°C at 1300 nm. The proposed fiber device could potentially constitute an efficient route towards realization of monolithic tunable fiber filters or sensing elements.

Since the first experimental report in 1996 1, photonic crystal fibers (PCFs) have had a significant impact in the fiber-optic community attracting the attention of several research groups around the globe. PCFs can be fabricated from a single material which can be either polymer^2^ or glass^1^, with silica being the most perhaps widely used and mature optical fiber material. They are usually constituted from a solid-core and a hexagonal array of holes running along the entire length of the fiber^1^. The unique ability to tailor and control the light based on the geometrical characteristics of the PCF, opened the door to the exploitation of new fundamental optical phenomena^3^ and applications^4^ using both solid and hollow-core antiresonant fibers^5^,^6^,^7^. Another interesting feature of these fibers is that they can act as “substrate” able to host novel functional optical materials inside their cladding air-holes^8^. Infiltration of advanced active materials such as liquid-crystals^9^,^10^, polymers^8^,^11^,^12^,^13^, high-index liquids^14^,^15^, metals^16^,^17^, bio-layers^18^,^19^, glasses^20^,^21^, etc. into the periodic cross sectional air-hole array of the PCF, allowed the development of advanced fiber devices that can act as switches^9^,^10^, filters^15^,^16^,^17^,^20^,^21^, tunable components^11^,^12^,^13^ and sensors^11^,^12^,^13^,^14^,^15^. There are limited reports so far demonstrating the combination of silica PCFs with chalcogenide glasses for the development of tunable bandgap devices. Granzow *et al*. reported the infiltration of chalcogenide glass in the holes of PCF by applying high pressure at ~660 °C^22^. The infused high index glass introduced photonic bandgap guidance. However, it was not demonstrated whether the photonic band gap can be tuned. The proposed method is an efficient and straight forward way of PCF infiltration with molten glass but specialized and expensive custom-made equipment is required^22^. In 2012, it was reported for the first time a new facile and cost-effective method of depositing chalcogenide glass films inside PCFs by using a solution-derived approach requiring ambient conditions and no sophisticated equipment^23^. One of the most important advantages of that approach was the possibility to modify the guidance mechanism of the light from index-guiding to anti-resonant reflecting optical waveguide (ARROW) with only a few nanometers thick high-index glass films with the air-holes be still available for further functionalization or post-processing^23^,^24^,^25^.

Chalcogenide glasses have existed for more than 60 years, yet they have only recently been proven as emerging materials offering new possibilities in photonics^26^. Their most important optical properties are their extremely high non-linear coefficients^26^, high refractive indices n = 2–3.5 26, photo-tunability properties when illuminated with light at wavelength near their band-gap edge^26^ and their ability to be transparent in the infrared regime^27^. Solution-processed chalcogenide glasses were first investigated by Chern *et al*.^28^. Nowadays, this method has attracted increasing interests in the optics community, mainly for different applications such as chemical sensors, data storage devices, lenses and infrared detectors^29^,^30^,^31^,^32^. The solution-processed approach of chalcogenide glass has discrete advantages over other deposition techniques such as simple preparation procedure, deposition of thin or thick films on complex and non-planar structures, integration among different optical devices, etc.^30^. Furthermore, this method can be adopted for any chalcogenide glass composition and can be expanded to any solution-dissolved material or even for stacked multi-material layer deposition. In this work, we experimentally demonstrate for the first time that ultra-thin stoichiometric high-index As_2_S_3_ films can be used to thermally tune the antiresonant windows in silica PCF due to their high thermo-optic coefficient^33^. We used two different commercially available silica PCFs infiltrated with the solution-processed glass. After certain annealing treatment of the solution-infiltrated fibers, nanometer-scale glass layers formed in the inner surface of the air-holes of the PCF introducing strong as high as ~40 dB resonances from visible up to near-infrared wavelength range. We show that the high thermo-optic coefficient 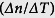 of the chalcogenide films allows the tuning of the transmission band red-edges as high as ~170 nm over 48 °C.

## Results

### Material and sample preparation

**Figure 1:**
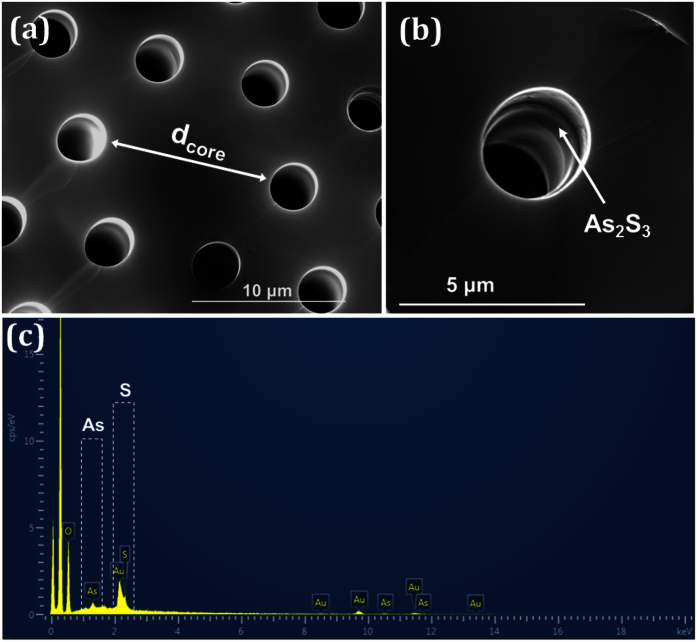
Scanning Electron Microscope image of (**a**) the LMA-10 PCF infiltrated with the As_2_S_3_ films and (**b**) a single magnified hole showing the thin deposited film. (**c**) Energy dispersive X-ray spectroscopy verifying the existence of the Arsenic and Sulfide elements on the inner wall of the air-holes.

For our experiments, we used two different commercially available PCFs (NKT Photonics A/S) named as LMA-5 and LMA-10 with core diameters *d*_*core*_, 5 and 10 μm and cladding air-hole diameters *d*, 1.3 and 3.5 μm, respectively. Both PCFs have a relative hole size *d*/*Λ* < 0.42 where *d* is the air-hole diameter and *Λ* the distance between the holes, ensuring thus they are endlessly single-mode^34^. After the preparation of the nanocolloidal As_2_S_3_ chalcogenide glass solution in ethylenediamine (EDA) (see Methods), the low-viscosity material was infiltrated in both PCFs using the capillaries forces at room temperature. The viscosity of the dissolved As_2_S_3_ in EDA as well as in n-propylamine or n-butylamine shows Newtonian behavior and increases with concentration^35^. The glass concentration prepared in our case was 550 mg/mL. The properties of the glass solution do not depend on whether crystalline or amorphous material is used^35^. Figure 1(a) shows the Scanning Electron Microscope (SEM) image of the core area of the hybrid LMA-10 PCF with the high index films and Fig. 1(b) the formed thin chalcogenide glass layers of As_2_S_3_ at the inner surface of a single hole. The formed glass films are relatively uniform along the fiber as it has been experimentally demonstrated elsewhere^23^,^36^. The thickness of the chalcogenide films in this case could not be accurately determined, since it was only a few nanometers thick. In order to verify the presence of the formed glass, Energy Dispersive X-ray (EDX) spectroscopy was applied for element analysis. Figure 1(c) shows the recorded EDX spectrum confirming the existence of the As (arsenic) and S (sulfide) lines. The LMA-5 hybrid PCF was also prepared in a similar manner (see Supplementary Information Fig. S1).

**Figure 2:**
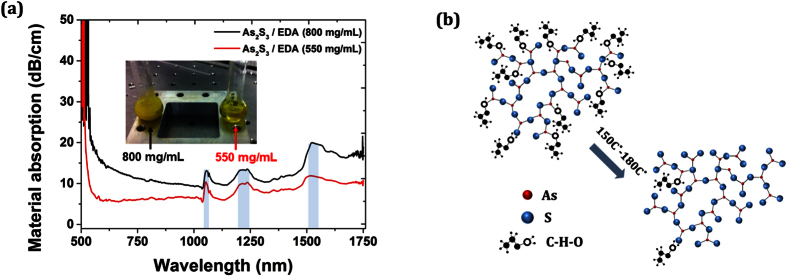
(**a**) Absorption spectra of two solution processed As-S based chalcogenide glasses dissolved in EDA. Inset: pictures of the two solutions used in our absorption experiments. The highlighted blue strips indicate the absorption bands arising from the solvent. (**b**) Illustration of the As_2_S_3_/EDA network and how the solvent molecules are removed as annealing temperature increases. The red and blue spheres correspond to As and S, respectively while the black and circle connected dots corresponds to C-H-O bonds.

The concentration of the chalcogenide solution plays a crucial role in the formation of the final thickness of the glass film. It has been reported that as the glass concentration (mg/mL) increases, the thickness of the formed glass inside the air-holes of the PCF increases to a certain extend^23^,^36^. Importantly, the glass concentration of the solution defines the loss of the material. In order to investigate how the concentration affects the absorption properties of the solution, we prepared two different As_2_S_3_ glass solutions dissolved in EDA with concentration 550 and 800 mg/mL as shown in the inset image of Fig. 2. For the high concentration solution (800 mg/mL) the amber yellow color is denser with limited transparency compared to the low concentration (550 mg/mL) solution. The absorption spectra of the two materials were measured using a liquid cell and a supercontinuum source (see Methods). The loss profile of the concentrations is shown in Fig. 2(a). For the high concentration solution (800 mg/mL), the absorption is significantly higher compared to the low concentration solution. Since the solution is based on the chalcogenide glass and the organic solvent (EDA), the absorption characteristics of the two are expected to be different. The chalcogenide As_2_S_3_ glass exhibits high absorption as the spectrum range moves from infrared towards visible wavelengths and close to the bandgap of the material (~2.52 eV). On the other hand, the organic solvent has high transparency in the visible range, while distinct absorption bands appear in the near-infrared region. We individually measured the absorption spectrum of EDA verifying the absorption lines observed at ~1050, ~1235 and ~1560 nm shown in Fig. 2(a) (see Supplementary Information Fig. S2). Similar to EDA, other amine-based solvents have been used in the past to derive solution-processed chalcogenide glasses such as n-butylamine and n-propylamine^30^,^35^. A direct comparison of the absorptioln spectra of these three widely used solvents is also provided (see Supplementary Information Fig. S2).

The dissolution process and kinetics in a hybrid chalcogenide glass-amine system was first described by the Chern *et al*.^28^,^37^,^38^. They proposed that the organic solvent breaks up the bulk amorphous glass into small flat clusters if we treat the initial bulk material as layer-like structure based on the nucleophilic substitution of a sulfide atom by the alkyl amine group of the n-butylamine solvent. In late 1980’s, Guiton and Pantano investigated the dissolution of arsenic sulfide in a different amine solvent, ethylenediamine (EDA), as we also used in this study^35^. They reported that the formation of alkyl-ammonium salts or hydrogen sulfide in this system does not occur as with n-butylamine but instead polymer-like chains of As_4_S_4_ rings formed and interlinked by bridging sulfur atoms with each ring chelated by two solvent molecules. This completely different mechanism is only possible due to the chelating nature of a diamine solvent^30^,^35^.

By increasing the temperature, the solvent starts evaporate making thus the solution more viscous and the process is then similar to the sol-gel process of silicate gels. After the infiltration of the solution into the capillaries of the PCF over ~6 cm, two stage annealing process was applied. First, we soft-annealed the sample at low temperatures to remove most of the solvent and we further annealed the sample close to the glass transition temperature (Tg) of the chalcogenide to remove the residual solvent and densify the glass. Figure 2(b) shows a schematic representation of the amorphous glass/solvent network and how the solvent molecules are drastically removed from the glass network as the temperature increases close to the T_g_ of the bulk glass.

### Optical characterization

We characterized the hybrid As_2_S_3_/silica PCF using a high power supercontinuum laser source covering from 480 up to 2200 nm (see Methods). The light was coupled into the fiber while the near-field output was recorded using a high resolution CCD camera. Finally the spectrum was recorded using a large core multimode silica fiber and a high resolution optical spectrum analyzer (OSA).

**Figure 3:**
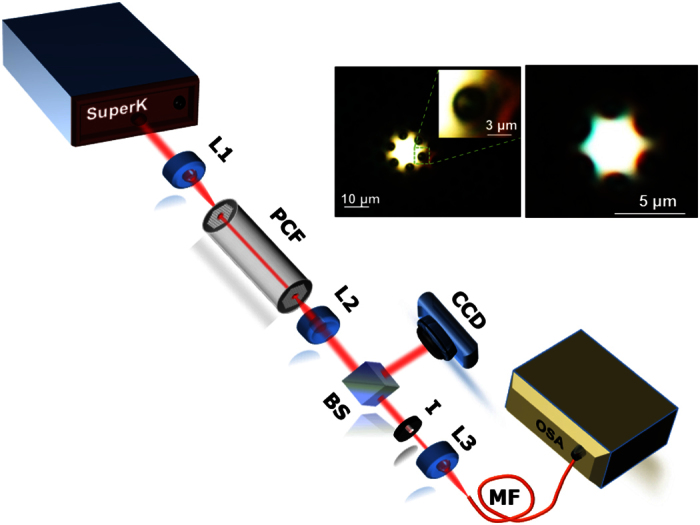
Experimental set up. SuperK: supercontinuum laser source, L1, L2, L3: microscope objective, BS: Beam splitter, I: Iris diaphragm, MF: multimode fiber.

The characterization set up used in our experiments is shown in Fig. 3 while the two images show the near-field profile for the two cases of LMA-10 and LMA-5. From the near field images it can be also seen that small fraction of light has been coupled to the high-index films.

**Figure 4:**
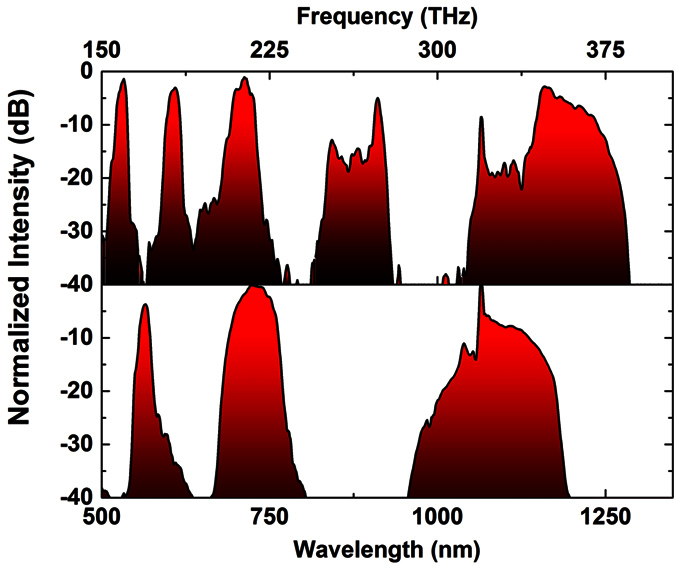
Transmission spectrum of the hybrid As_2_S_3_/silica LMA-10 (top) and LMA-5 (bottom) PCF showing distinct resonances from 500 nm up to 1350 nm (top axis corresponds to frequency).

The deposited high refractive index nanofilms introduce distinct resonances in the transmission spectrum of the hybrid PCF as the guiding mechanism is no more based on total internal reflection. This guiding mechanism has been extensively described using the ARROW model. The ARROW model first introduced in 1986 describes how the light is confined in a planar silicon waveguide having as cladding a number of low and high index layers^39^. Later in 2002, Litchister *et al*. explained that the same principal can be used to describe the light transmission not only in planar multi-layer structures but also in solid-core fibers with high index inclusions as well as bandgap fibers^40^. Same mechanism applies also in our hybrid fibers in which each high refractive index layer can be considered as a Fabry–Perot (F-P) resonator. For on-resonance state, the F-P cavity is transparent allowing the light to escape from the structure, while the reflectivity can be very high for off-resonance state strongly confining the light in the core of the fiber. The locations and the bandwidth of the resonant windows are defined mainly by the refractive index and the thickness of the high-index chalcogenide glass nanofilms^23^,^36^. Since the glass nanolayers were derived from a solution, the exact refractive index value is not known. Figure 4 shows the transmission spectra of the LMA-10 (top) and LMA-5 (bottom) with As_2_S_3_ glass films. In both cases, resonances with extinction ratio (defined as maximum to minimum transmission) as high as ~40 dB can be clearly seen. As_2_S_3_/LMA-10 PCF revealed 5 different resonance windows with each one having a central wavelength at around 532, 610, 711, 880 and 1150 nm. It is pertinent to note that, the hybrid LMA-10 PCF is not single mode and therefore the fine structure and features appear in the spectrum are mainly introduced from the interference between the higher order modes. Similarly, As_2_S_3_/LMA-5 PCF revealed three different resonances at 563, 727 and 1084 nm. It should be noted that for both cases, as the wavelength increases, the bandwidth of the transmission window increases as well, as expected based on the ARROW model^23^,^36^,^40^.

The main property of chalcogenide glasses perhaps is their transparency in the mid-IR region due to their low phonon energy combined with their extremely high nonlinearity which can be even 1000 times higher than silica^26^,^27^. However, another important property chalcogenide glasses possess is their high thermo-optic coefficient 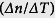
33. The thermo-optic coefficient of chalcogenide glasses can be significantly higher than silica allowing the development of thermo-tunable planar devices^33^. Here we demonstrate for the first time to the best of our knowledge that this property can be also used to tune the resonant windows in a PCF coated with nanometers thick chalcogenide glass layers.

**Figure 5:**
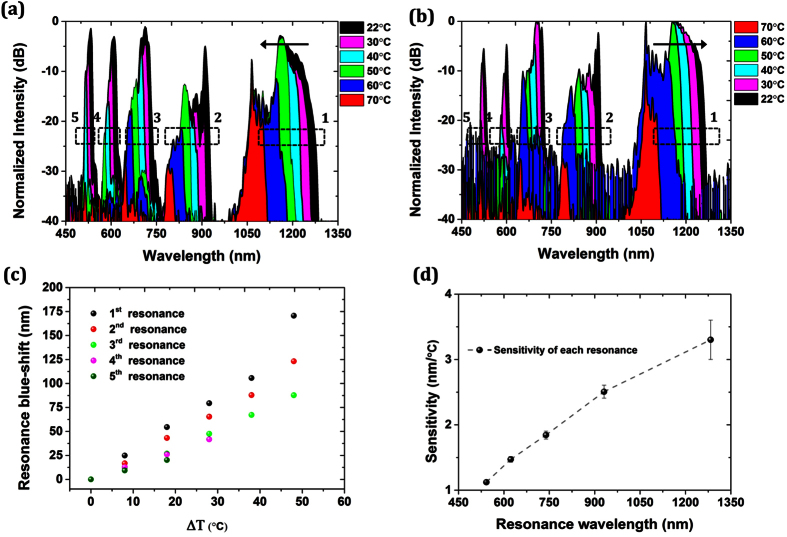
Thermal response of the hybrid LMA-10 As_2_S_3_/silica PCF (**a**) as the temperature increases and (**b**) decreases from 70 °C to room temperature. (**c**) Blue-shift of every resonance as temperature increases (spheres in different color represent every resonance). (**d**) Sensitivity (defined as nm resonance shift/°C) for every resonant wavelength.

Figure 5(a) shows the blue-shift of the narrow long-edge resonant windows of the hybrid LMA-10 PCF as temperature increases from 20 °C up to 70 °C. Every resonant edge, with the first one be at ~1300 nm, has been marked with a dotted window for clarity purposes. As temperature increases the transmission edge is shifting to shorter wavelengths. Small variations of the transmission spectrum are attributed to the interference of the higher-order modes combined with the intensity fluctuations of the source (see Supplementary Information Fig. S3). Interestingly, chalcogenide glasses can have both negative and positive thermo-optic coefficient based on the glass composition and the operating wavelength^33^. This ability further extends their flexibility to be synthesized and used according to the desired application every time. Since here the negative thermo-optic coefficient of the chalcogenide glass at short wavelengths^33^,^41^ is larger than the positive thermo-optic coefficient of silica, the temperature-induced change in refractive index of chalcogenide glass (e.g. 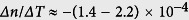 for As_2_S_3_ at 810 nm^41^) is dominating over the very small 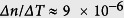 at 1550 nm^42^ of the host PCF material. An increase in temperature will therefore decrease the refractive index contrast between the host silica material and both the chalcogenide glass films and the average cladding-hole index. This change in the refractive index contrast directly influences the transmission bands by blue-shifting their location ~200 nm at 1100 nm for example when the temperature increases from 22 °C to 70 °C. Similar blue-shift behavior, based on other active materials with negative thermo-optic coefficient, has been also experimentally reported elsewhere^43^,^44^.

Figure 5(b) shows the transmission spectrum of the hybrid PCF as temperature decreases. It is evident that now the long-edge resonance is red-shifting as temperature decreases as expected. We repeated the measurements over two full temperature cycles (forward and reverse) in order to verify the repeatable response of the hybrid PCF (see Supplementary Information Fig. S4). Figure 5(c) shows the corresponding shift of every resonance over a temperature range Δ*T* ∼ 50 °*C*. At long wavelengths, the blue-shift is larger compared to the short wavelengths. This is because the fundamental guiding mode expands further in longer wavelengths enhances thus the light-chalcogenide interaction. By defining the sensitivity of the hybrid fiber as nm shift of the resonance per °*C* at every resonant wavelength starting from ~600 nm, it was revealed a sensitivity as high as ~3.6 nm/°C at ~1300 nm as shown in Fig. 5(d). This sensitivity is comparable or even higher than several other reports on liquid or solid filled-PCFs^45^,^46^, while the air-holes in our case are still available for further post-processing.

**Figure 6:**
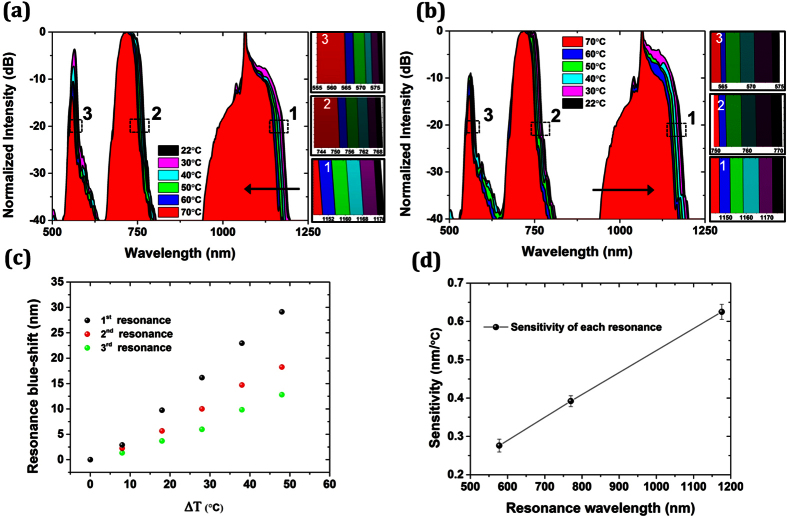
Thermal response of the hybrid LMA-5 As_2_S_3_/silica PCF (**a**) as the temperature increases and (**b**) decreases from 70 °C to room temperature. (**c**) Blue-shift of every resonance as temperature increases (spheres in different color represent every resonance). (**d**) Sensitivity (defined as nm resonance shift/°C) for every resonant wavelength.

Similarly we investigated the thermal response of the hybrid LMA-5 PCF. The deposition of the high index glass films inside the holes of the PCF introduced three main transmission windows in this case with central wavelengths ~563 nm, ~727 nm and ~1075 nm as shown in Fig. 6(a). Increasing the temperature from 22 °C to 70 °C, the transmission resonances are also blue-shifting. However, the shift in this case is smaller compared to the LMA-10 PCF. This is because the fiber in this case supports only the fundamental mode. On the other hand, LMA-10 can support more than one mode and because the higher-order modes can have higher intensity at the silica-chalcogenide glass interface where the nanolayers are located, the sensitivity of LMA-10 is higher than that of LMA-5. The resonant long-edge shift of the transmission windows at ~1190 nm, 770 nm and 579 nm are indicated as 1, 2 and 3, respectively. The side color bars images are the zoomed-in sections of the marked areas (i.e. 1, 2 and 3) in Fig. 6(a) indicating the blue-shift of the long-edge of the band. We also repeated the measurements as the temperature decreases, i.e. from 70 °C to 22 °C. The resonances were red-shifted returning to their initial location as shown in Fig. 6(a). Tracking the long wavelength resonance at ~1190 nm, the sensitivity was found as high as 0.625 nm/°C (Fig. 6(d)) which is almost 6 times lower than LMA-10 PCF. It should be noted that the sensitivity can be further increased in both cases (LMA-10 and LMA-5 PCF) by properly tailoring the glass composition^33^ and optimizing the design of the hybrid chalcogenide/silica PCF.

## Discussion

In summary, we have experimentally demonstrated the possibility to develop a thermo-tunable hybrid PCF based on solution-processed chalcogenide glass nanolayers using two different commercially available silica PCFs. The high index layers modified the guiding mechanism of the fiber from index- to ARROW guidance and we show how the revealed transmission windows can be thermally tuned from 22 °C to 70 °C due to the high thermo-optic coefficient of chalcogenide materials. The developed fiber devices exhibited consistent and repeatable response and their sensitivities were found ~0.625 and ~3.6 nm/°C with LMA-5 and LMA-10 PCF, respectively. Furthermore, the air-holes of the proposed hybrid PCFs are still available for further functionalization or even for multi-material deposition. The great flexibility and unique optical properties offered by chalcogenide glasses combined with silica PCFs open a new highway towards all-fiber tunable devices and probes.

## Methods

### Nanocolloidal solution-based chalcogenide glass preparation

High purity (99.999%) amorphous As_2_S_3_ bulk pieces were purchased from Alfa Aesar. The bulk As_2_S_3_ glass was grinded into fine powder using a ceramic mortar in an N_2_ environment and then dissolved in ethylenediamine (EDA) (purity >99%) inside a sealed glass flask to stop any solvent evaporation. A magnetic stirrer was used to expedite the dissolution process. The dissolution procedure required a few hours in order to ensure complete dissolution of the bulk glass. It should be emphasized that the absorption loss of the glass solutions could be further improved by using ultra-high purity chalcogenide glass prepared based on the melt-quenching technique. The hybrid PCFs were infiltrated and rested at room temperature for 48 h and then annealed at ~65 °C for 12 h. We further annealed the fibers at 170 °C for 48h in order to minimize the presence of the solvent and leave only the glass layer.

### Absorption Measurements

The absorption measurements were performed using a high power supercontinuum laser source (SuperK Versa, NKT Photonics A/S) with average power ~1.5 W over the 480–2200 nm wavelength region. Two different fused silica cuvettes of 1.4 mL were filled with the two As_2_S_3_/EDA glass solutions. A linearly variable optical attenuator placed between the laser beam path and the cuvette to control the incident power. The collimated beam from the supercontinuum laser was transmitted through the cuvette and the output light was recorded using an integrating sphere (Thorlabs) connected to the Optical Spectrum Analyzer (Ando AQ6317B) with minimum resolution 0.05 nm. The recorded spectra were normalized to the reference spectrum.

### Optical Characterization

The optical characterization of the hybrid As_2_S_3_/silica PCFs was made using the SuperK Versa (see specifications above). The light was coupled into the fiber using a 40x microscope objective (L1). The output beam was collimated with another 60x microscope objective (L2) and focused into a multimode fiber (60 μm core size) using a 10x microscope objective (L3). The signal was finally recorded with the OSA (Ando AQ6317B). All the undesired light was blocked by using an iris at the output in order to record only the light of the core. A beam splitter (BS) was also inserted between the output and the optical spectrum analyzer to capture the near-field profile of the fundamental core mode and the high-index cladding modes with a high resolution CCD camera. For the temperature measurements presented in Figs 5 and 6 in the manuscript, a controlled heating element (Linkam MC60) was placed in contact with the fiber.

## Additional Information

**How to cite this article**: Markos, C. Thermo-tunable hybrid photonic crystal fiber based on solution-processed chalcogenide glass nanolayers. *Sci. Rep.* **6**, 31711; doi: 10.1038/srep31711 (2016).

## Supplementary Material

Supplementary Information
