# Supplementary material for: Thermo-tunable hybrid photonic crystal fiber based on solution-processed chalcogenide glass nanolayers
Source: Sci Rep. 2016 Aug 19;6:31711. doi: 10.1038/srep31711 (PMC4990916; doi:10.1038/srep31711)
Supplement: Supplementary Information [file srep31711-s1.pdf]

Supplementary Information:

Thermo-tunable hybrid photonic crystal fiber based on solution-processed chalcogenide glass nanolayers

Christos Markos

DTU Fotonik, Department of Photonics Engineering, Technical University of Denmark, DK-2800 Kgs. Lyngby, Denmark

Scanning Electron Microscopy

After the chalcogenide glass integration inside the holes of the silica PCFs, the end facets were cleaved using a ceramic cleaving tile. The SEM images in every case were taken with a *FEI Quanta 200 ESEM FEG Electron Microscope* using an accelerating voltage of 2-20 kV combined with energy dispersive X-ray Spectroscopy (EDX) (using an Oxford Instruments 80 mm^2 X-Max silicon drift detector) in order to confirm the existence of the two main elements of the chalcogenide nanofilms, Arsenic (As) and Sulfide (S). In addition to Fig. 1 in the manuscript, we imaged the hybrid LMA-5 PCF after the glass deposition and post-annealing treatment to confirm the presence of the chalcogenide glass films in the holes of the fiber. Figure S1 (a) shows the initial LMA-5 silica PCF used in our experiments and Fig. S1 (b) shows the core section of the fiber after integration of As_2S_3 glass indicating the formation of the glass nanolayers in the air-holes of the fiber. It should be noted that Fig. S2 (b) is slightly distorted as the angled cleave of the fiber introduced strong electron charging inside the holes of the fiber (white areas).

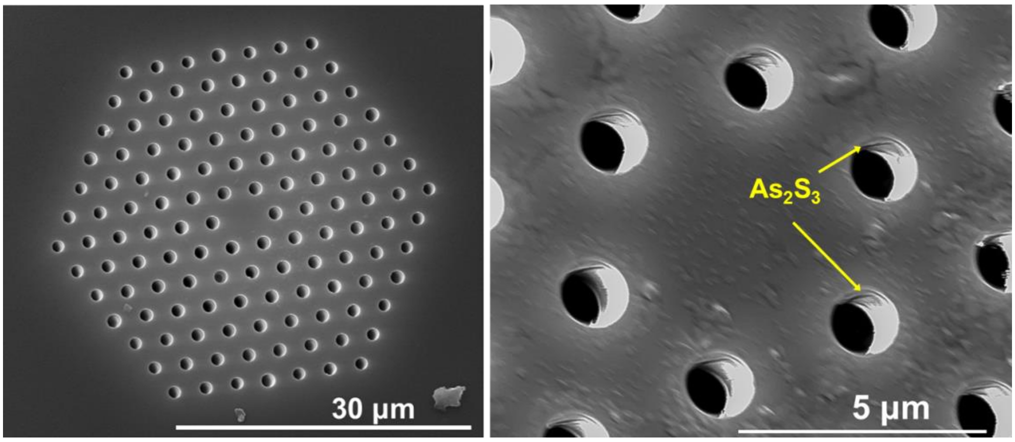

Figure S1: (a)Scanning Electron Microscope image of the initial LMA-5 silica PCF and (b) after the deposition of the As_2S_3 glass nanolayers.

Amine solvent absorption measurements

The most widely amine solvents used for dissolving chalcogenide glass are n-butylamine, n-propylamine and ethylenediamine (EDA). As the dissolution kinetics form a glass-solvent network, the solvent itself contributes to the total absorption of the solution. Figure S2 shows for the first time (to the best of our knowledge) the absorption spectra of the three amine

solvents (n-butylamine, n-propylamine and ethylenediamine) used to develop dissolved-derived chalcogenide glasses from 550 nm up to 1750 nm. The measurements were performed using a liquid cell (cuvette), a supercontinuum source and an integrating sphere similar to the configuration used for the measurement in Fig 2 (a) (see Methods). EDA has the strongest absorption peak at ~1050 nm compared to n-butylamine and n-propylamine. However, the main advantage of EDA is that homogeneous dissolution of the bulk As_2S_3 glass can be achieved within a few hours while n-butylamine and n-propylamine require several weeks under stirring. Furthermore, the three solvents are highly transparent in the visible range (550 – 750 nm) and therefore their loss levels are close to the background noise as shown in Fig. S2.

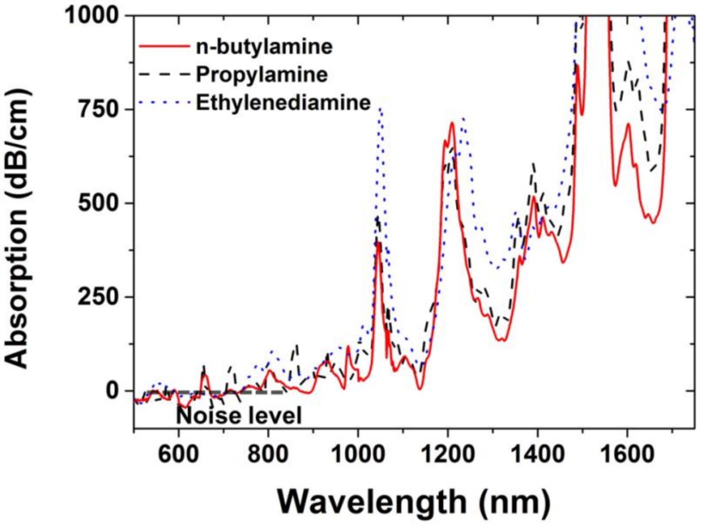

Figure S2: Absorption profile from 550 nm up to 1750 nm of n-butylamine (red-solid line), n-propylamine (black-dashed line) and ethylenediamine (blue-dotted line).

Power stability and device repeatability measurements

For our experiments we used a commercially available supercontinuum source (SuperK Versa). We measured the intensity stability of our source versus time. Figure S3 shows that the power of the source is not fully stable over time (5 hours). This can explain the small spectral

variations of Fig. 5 (a) and (b) which they are introduced mainly due to interference of the higher order modes combined with the intensity fluctuations of the source.

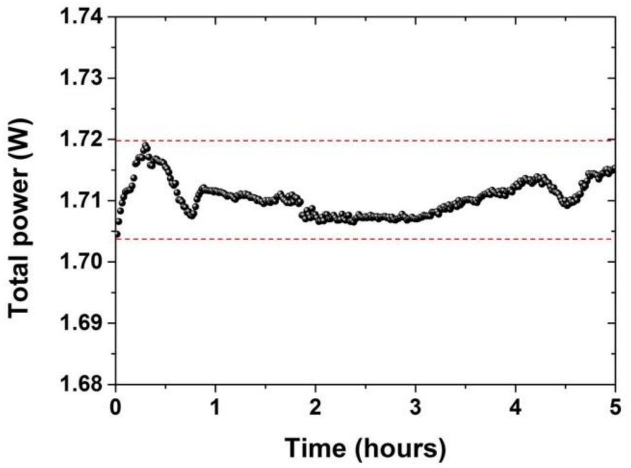

Figure S3: Intensity characterization of the SuperK Versa supercontinuum source (NKT Photonics) over 5 hours using a thermal head detector, a power meter and a computer.

The repeatability of the hybrid LMA-10 device was tested by heating and cooling the fiber over two full cycles. The following Fig. S4 (a) and (b) shows the long-edge response of the fiber by tracking the resonance at ~1300 nm.

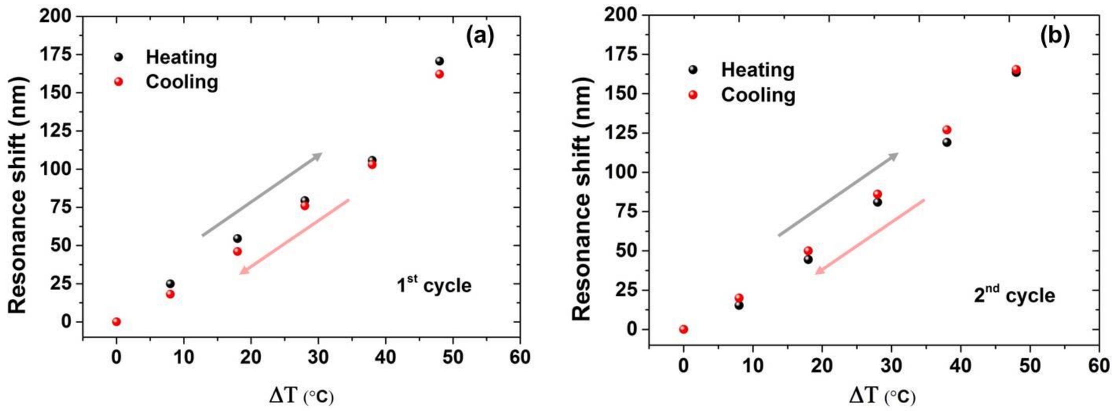

Figure S4: Resonance shift of the hybrid LMA-10 As_2S_3/silica PCF at ~1300 nm for (a) the 1st full heating/cooling cycle and (b) 2nd full heating/cooling cycle.
